# The Mediating Role of Psychological Capital in the Relationship Between Transformational Leadership and Professional Identity Among Chinese Anesthesia Nurses

**DOI:** 10.1155/jonm/8250599

**Published:** 2026-09-14

**Authors:** Rui Gao, Peishuang Wang, Yi Yang, Fang Zhou, Jiao Wu, Nana Liu, Huihui Hu

**Affiliations:** ^1^ School of Nursing, Xuzhou Medical University, Xuzhou, Jiangsu, China, xzmc.edu.cn; ^2^ Department of Anesthesiology Operating Room, Affiliated Hospital of Xuzhou Medical University, Xuzhou, Jiangsu, China, xzmc.edu.cn

**Keywords:** anesthesia nurses, mediating effect, professional identity, psychological capital, transformational leadership

## Abstract

**Background:**

Professional identity is crucial to clinical work quality and the career development of medical staff. With the rapid development of anesthesia nursing in China, identifying factors that improve anesthesia nurses’ professional identity carries important practical implications for the long‐term sustainable development of the anesthesia nursing profession.

**Aims:**

This study aimed to investigate the current level of professional identity among Chinese anesthesia nurses, examine the relationship between transformational leadership and professional identity, and verify the mediating role of psychological capital in this relationship.

**Methods:**

Using convenience sampling, anesthesia nurses from tertiary hospitals in nine provinces and municipalities were recruited from March to June 2024. Data collection involved the General Information Questionnaire, the Transformational Leadership Scale, the Psychological Capital Scale, and the Professional Identity Scale. Path analysis was conducted using AMOS 28.0 software to establish structural equation models. The study was reported following the STROBE checklist.

**Results:**

The total scores for transformational leadership, psychological capital, and professional identity were 107.63 (SD = 18.96), 97.59 (SD = 14.06), and 37.18 (SD = 7.97), respectively. The results showed a positive correlation among transformational leadership, psychological capital, and professional identity (all *p < *0.01). Additionally, psychological capital was identified as a partial mediator between transformational leadership and professional identity, accounting for 44.06% of the total effect.

**Conclusion:**

Anesthesia nurses exhibited a moderate level of professional identity. Additionally, psychological capital acted as a partial mediator between transformational leadership and professional identity.

**Implications for Nursing Managers:**

Nursing managers are recommended to adopt a transformational leadership style and strengthen psychological capital interventions, which can further enhance the professional identity of anesthesia nurses, thereby stabilizing the anesthesia nursing workforce, improving the quality of clinical care, and ultimately promoting the sound development of the anesthesia nursing profession.

## 1. Introduction

With the promulgation of the “Notice on the Management of Anesthesia Outpatient Departments and Nursing Units in Medical Institutions” [1] and the “Notice on Issuance of Opinions on Strengthening and Improving Anesthesia Medical Services” [2], the profession of anesthesia nursing has emerged in China. Although anesthesia nursing in China is still in its infancy compared to more developed regions such as Europe and the United States, it possesses enormous development potential and promising career prospects. As the core practitioners in anesthesia nursing, anesthesia nurses play a crucial role in enhancing the efficiency of surgical and anesthesia procedures, ensuring patient safety, improving patient comfort, and elevating the quality of anesthesia care services. Therefore, their professional identity within anesthesia nursing serves as a vital safeguard for the healthy development of this emerging profession.

## 2. Background

Professional identity [3] refers to an individual’s positive cognition and emotional investment in their profession, including the recognition of the values of work and the commitment to responsibilities. In nursing, professional identity reflects the extent to which nurses’ recognition of the intrinsic value of their profession, its social significance, and their professional responsibility [4]. It is the bedrock of nursing practice and the progression of nurses’ careers. Positive professional identity not only enhances nurses’ subject motivation and job satisfaction but also strengthens their professional confidence and self‐esteem [5]. Moreover, it improves nursing quality and teamwork skills, which are essential for stabilizing the nursing workforce [6, 7]. Karin [8] emphasized that the professional identity of anesthesia nurses serves as the core for balancing technical operation with humanistic care, reducing role conflicts within interdisciplinary teams, alleviating burnout, and enhancing resilience. It is essential for establishing the unique value of the specialty and promoting professional development. However, as an emerging professional group, the professional identity of anesthesia nurses has not yet received sufficient attention. Considering that anesthesia nursing in China is at a pivotal stage of development, exploring the professional identity of anesthesia nurses holds significant practical importance for defining their professional roles, optimizing clinical care quality, and supporting the long‐term growth of anesthesia nursing.

The professional identity of anesthesia nurses is related to various factors, including personal characteristics (e.g., age and specialization), work‐related aspects (e.g., occupational pressure) [9], and societal perceptions (e.g., others’ evaluations) [10]. Among organizational factors, leadership style [11] has been identified as a key factor influencing nurses’ professional identity. In particular, transformational leadership excels at creating a positive and supportive environment, making it the preferred choice for nursing managers [12]. It refers to leaders establishing common ideals and values with subordinates and motivating subordinates to fully realize their potential by means of empowerment and development to enhance nurses’ intrinsic motivation [13]. Its core connotation is reflected in four dimensions: moral modeling, inspirational motivation, charisma, and individualized consideration. By reshaping individual cognition and behavioral orientation, transformational leadership encourages team members to internalize organizational goals, thereby forming collective identity and team trust [14, 15]. Susana et al. [16] contended that transformational leadership, as an effective and proactive leadership style, serves as an essential external resource in nursing practice. According to the basic assumptions of the conservation of resources (COR) theory [17], individuals strive to acquire, retain, cultivate, and protect the resources their valued resources. Nurses can proactively acquire and continuously accumulate psychological, professional, and environmental resources through transformational leaders’ value guidance, empowerment incentives, and emotional support [18], thereby fulfilling their needs for resource acquisition and preservation. These positive resources stimulate nurses’ work passion, enhancing their sense of professional value and identity. Chen [19] also found that transformational leadership, as a management concept suited to China’s healthcare environment, positively correlates with nurses’ professional identity. Over the past decade, anesthesia nursing management in China has evolved into a specialized system led by anesthesia nursing team leaders and head nurses [20, 21], promoting the development of anesthesia nursing toward greater specialization and refinement. In this process, the leadership style of anesthesia nursing managers plays a crucial role in team growth and in shaping nurses’ professional attitudes. However, there are currently no clear reports on the level of transformational leadership among anesthesia nursing managers in China or on whether it influences the professional identity of anesthesia nurses.

For positive change to occur, it needs to be mediated by positive factors within the individual [22]. Psychological capital is an important internal resource of individuals, which includes self‐efficacy, hope, resilience, and optimism [23]. Ren et al. [24] discovered that psychological capital helps nurses maintain positive perceptions and identification with their profession by buffering resource depletion caused by occupational adversity. A study of nursing students indicated [25] that when confronted with complex work environments and challenges, those with higher psychological capital exhibit greater positive emotions, stronger coping abilities, and intrinsic motivation, thereby fostering a steadfast professional identity. Self‐efficacy is defined as an individual’s confidence in facing great challenges and overcoming difficulties [26]. The higher an individual’s self‐efficacy, the more likely they are to identify with their value and abilities in the professional field [27]. Therefore, psychological capital plays a positive role in promoting professional identity. In addition, a study in South Korea pointed out [28] that transformational leaders can enhance nurses’ psychological capital through pathways such as imparting professional meaning, providing care, and offering career development guidance. Sun [29] also found that the stronger the transformational leadership capabilities of head nurses, the greater the nurses’ latent inner strength and psychological capital. According to the COR theory [17], individuals achieve resource enhancement spirals by acquiring and accumulating resources, which promotes individual growth. When nurses have transformational leadership as a positive external resource, their internal psychological energy is stimulated, and their psychological capital is increased. This achieves resource value enhancement and strengthens their professional identity. However, research is lacking on whether psychological capital mediates the relationship between transformational leadership and professional identity among anesthesia nurses.

**FIGURE 1:**
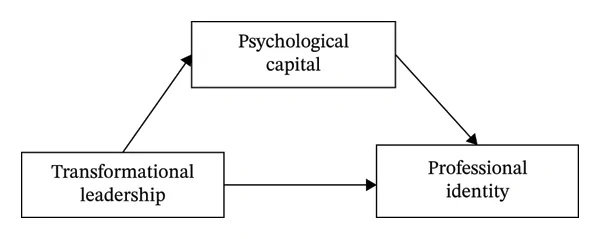
The hypothetical model diagram.

In summary, this study aims to investigate the level of professional identity among anesthesia nurses, the level of transformational leadership exhibited by anesthesia nursing managers, and the relationship between psychological capital and these two factors during the early developmental phase of anesthesia nursing in China. The findings may potentially further compensate for the limitations in current research on anesthesia nurses and enrich the application of COR theory in the field of anesthesia nursing. In addition, they could offer targeted guidance and scientific evidence for optimizing anesthesia nursing management, enhancing nurses’ professional identity, and promoting the high‐quality development of the anesthesia nursing profession. Based on previous research and grounded in COR theory, this study proposes the following hypotheses and constructs the relational model diagram depicted in Figure 1.

 H1: Transformational leadership is positively related to professional identity.
 H2: Psychological capital mediates the relationship between transformational leadership and professional identity.

## 3. Methods

### 3.1. Design

The cross‐sectional descriptive study was conducted from March to June 2024.

### 3.2. Setting and Sample

Considering the feasibility of the study, the current status of anesthesia nursing development in China, and the limited number of anesthesia nurses in various hospitals, this study employed convenience sampling to select anesthesia nurses from tertiary hospitals in nine provinces and municipalities with relatively well‐established anesthesia nursing units, including Beijing, Shanghai, Jiangsu, Anhui, Henan, Hubei, Zhejiang, and Shandong. Inclusion criteria: (1) Registered nurses with practicing qualifications; (2) Nurses who have been engaged in anesthesia nursing for 1 year or more; and (3) Voluntarily participate in this study. Exclusion criteria: (1) Anesthesia nurses who were not on duty during the study period; and (2) Anesthesia trainee nurses.

The sample size was calculated using G∗Power software. The moderate effect size was set at 0.15 and *α* at 0.05. After calculation, the required sample size was 238. Considering an invalid questionnaire rate of 20% and given that the sample size of the structural equation model needs to be ≥ 200, it was determined that the sample size for this study should be at least 298 cases.

### 3.3. Measures

The data were collected using self‐reported questionnaires, including the General Information Questionnaire, Transformational Leadership Scale, Psychological Capital Scale, and Professional Identity Scale. All tools employed in this study were Chinese versions, and detailed information about each scale is described as follows.

#### 3.3.1. General Information Questionnaire

Based on the purpose of this research and the review of previous literature, a general information questionnaire was designed, including demographic information and work situation. The demographic information included gender, age, marital status, and education level. Work status included employment category, professional title, monthly income, and years of work experience.

#### 3.3.2. Transformational Leadership Scale

The Transformational Leadership Scale was developed by Li et al. [30] based on the multifactorial leadership questionnaire developed by Bass and Avolio [13], combined with China’s national conditions and unique culture, in 2005. It is used to assess the degree to which employees perceive transformational leadership and has been widely applied among enterprise employees, teachers, medical staff, and other groups. This questionnaire consists of 4 dimensions and 26 items, namely moral modeling (8 items), inspirational motivation (6 items), individualized consideration (6 items), and charisma (6 items). It is rated on a 5‐point Likert scale, with scores ranging from “completely inconsistent” to “completely consistent” based on the level of self‐agreement. The higher the score, the higher the nurse’s perception of transformational leadership. The Cronbach’s *α* of each dimension of the scale ranged from 0.840 to 0.920. In this study, the Cronbach’s *α* was 0.985.

#### 3.3.3. Psychological Capital Scale

The Psychological Capital Scale was developed by Luthans et al. [31], localized by Li Chaoping [32], and adapted by Luo Hong [33] to evaluate the psychological capital level of nurses. It includes 4 dimensions: self‐efficacy (6 items), hope (6 items), resilience (5 items), and optimism (3 items). It is rated on a 6‐point Likert scale, with scores ranging from “*strongly disagree*” to “*strongly agree*” based on the level of self‐agreement. In this study, Cronbach’s *α* was 0.970.

#### 3.3.4. Professional Identity Scale

The Professional Identity Scale was jointly developed by Tyler and McCallum [34] and localized by Cai [35]. It is a single‐dimensional scale consisting of 10 items, used to assess an individual’s identification with their profession. It is rated on a 5‐point Likert scale, with scores ranging from “*strongly disagree*” to “*strongly agree*” based on the level of self‐agreement. The higher the score, the stronger the sense of professional identity. After converting the total score to a percentage, scores below 60% (less than 30) indicate low professional identity; scores between 60% and 80% (30–40) indicate moderate professional identity; and scores above 80% (greater than 40) indicate high professional identity [36]. In this study, Cronbach’s *α* was 0.947.

### 3.4. Data Collection

#### 3.4.1. Presurvey

From January to February 2024, researchers selected 36 anesthesia nurses from two tertiary hospitals who met the inclusion and exclusion criteria to conduct a presurvey. Based on the feedback collected from the respondents, the researchers revised ambiguous and unclear expressions in the general demographic data section and the instructions, and optimized the questionnaire completion process, thereby finalizing the official version for the formal survey. The purpose of this presurvey was to verify the comprehensibility of the questionnaire for anesthesia nurses, identify potential issues in data collection, and lay the foundation for the smooth conduct of the formal survey and ensure data quality.

#### 3.4.2. Formal Survey

Based on https://www.wjx.cn, a commonly used free online survey tool in China, this study uses survey links and two‐dimensional codes. After obtaining consent from the anesthesia care managers at each hospital, the researchers distributed the questionnaires via Wenjuanxing, and the research subjects gave informed consent. To ensure the authenticity and completeness of the data, each IP address was allowed to submit the questionnaire only once, and submissions were accepted only after all questions had been answered. To minimize nonresponse bias, participants received a monetary incentive in the form of a red envelope. To ensure the quality of the questionnaire, a response time standard of at least 5 min was set, and the completion time was critically reviewed by the research staff to ensure that the collected data had sufficient truth and reference value. A total of 354 questionnaires were distributed in this study. After eliminating 18 invalid questionnaires, 336 valid questionnaires were obtained, with an effective response rate of 94.9%.

### 3.5. Data Analysis

The qualified questionnaires were collected via Wenjuanxing, and the data were organized into an Excel table. The categorical variables of general demographic data were assigned and entered. Data analysis was conducted using SPSS Version 26.0 and AMOS Version 28.0. First, descriptive statistics and Pearson correlation analysis were conducted using SPSS 26.0 to summarize the data and examine relationships among variables. Normality was assessed by examining skewness and kurtosis. In this study, skewness of the variables was found to vary between −1.430 and −0.456, whereas the kurtosis values varied between 0.759 and 4.553. For normally distributed data, skewness within ±3 and kurtosis within ±10 were considered acceptable [37]. Subsequently, structural equation modeling (SEM) was performed using AMOS 28.0 to test the hypothesized relationships among variables. Maximum likelihood estimation and path analyses were employed to identify relationships among the three variables. Model fit was evaluated using the following criteria: *χ* ^2^/df < 3.00, RMSEA < 0.08, GFI > 0.90, CFI > 0.90, NFI > 0.90, TLI > 0.90. A *p* value of less than 0.05 was considered statistically significant. The bootstrap method was used to test the mediating effect.

### 3.6. Ethical Considerations

This research was approved by the Ethics Committee of Xuzhou Medical University (Approval Number: XZHMU‐2023611). All participants voluntarily participated in this study with a full understanding of the research content. Participants had the right to withdraw from the study at any time without any consequences. To ensure the privacy of the participants, all data were made available only to members of the research team. All procedures were performed in accordance with the principles of the Declaration of Helsinki.

## 4. Results

### 4.1. Common Method Deviation Inspection

The examination was conducted using the single‐factor confirmatory factor analysis (CFA). Specifically, all the observed variables involved in the study were loaded onto a single latent factor, and the overall fit of the hypothetical single‐factor model was then examined and compared with that of the original theoretical model. The results indicated that all the model fit indices failed to reach the acceptable threshold (*χ* ^2^/df = 36.993, CFI = 0.604, GFI = 0.461, AGFI = 0.192, NFI = 0.598), with the fitting effect being significantly inferior to that of the original theoretical model (*χ* ^2^/df = 3.909, CFI = 0.970, GFI = 0.919, AGFI = 0.869, NFI = 0.960). Therefore, there are no serious common methodological deviations in this study.

### 4.2. Participant Characteristics

A total of 336 anesthesia nurses participated in the study. Among them, 38 (11.3%) were men, and 298 (88.7%) were women. The participants had a mean age of 33.21 years (SD = 7.47). In terms of monthly income,51.2% reported that their income was between 8000 and 10,000 yuan. More than four‐fifths of the participants had a bachelor’s degree. The remaining demographic characteristics are detailed in Table 1.

**TABLE 1:** Demographic characteristics of participants (*n* = 336).

Variables	Category	*n*	%
Gender	Male	38	11.3
	Female	298	88.7

Age (years)	≤ 30	141	42.0
	31–40	136	40.5
	≥ 41	59	17.6
	Mean ± SD	33.21 ± 7.47	

Marital status	Single	100	29.8
	Married	236	70.2

Education level	High school or technical secondary	25	7.4
	Bachelor	297	88.4
	Master and above	14	4.2

Employment category	Permanent staff	90	26.8
	Contract system	187	55.7
	Personnel agency	59	17.6

Professional title	Junior	55	16.4
	Intermediate	111	33.0
	Senior	132	39.3
	Vice‐senior and above	38	11.3

Monthly income (RMB)	< 8000	142	42.3
	8000–15000	172	51.2
	> 15,000	22	6.5

Years of work experience(years)	≤ 5	57	17.0
	6–10	132	39.3
	≥ 11	147	43.8

*Note:* %, percentage; *n*, number.

Abbreviation: SD, standard deviation.

### 4.3. Descriptive Statistics and Correlation Analysis

As presented in Table 2, the score of the transformational leadership scale was 107.63 (SD = 18.96), wherein the moral modeling item scored the highest. The score of the psychological capital scale was 97.59 (SD = 14.06), wherein the self‐efficacy item scored the highest. The score on the professional identity scale was 37.18 (SD = 7.97).

**TABLE 2:** The scores of the transformational leadership scale, psychological capital scale, and professional identity scale of anesthesia nurses (*n* = 336).

Variables	Total score (Mean ± SD)	Average score of items (Mean ± SD)	Cronbach’s *α*	AVE	CR
Transformational leadership	107.63 ± 18.96	4.14 ± 0.73	0.985	0.815	0.946
Moral modeling	33.55 ± 6.16	4.19 ± 0.77	0.958		
Inspirational motivation	24.68 ± 4.75	4.11 ± 0.79	0.973		
Individualized consideration	24.53 ± 4.94	4.01 ± 0.82	0.975		
Charisma	24.88 ± 4.63	4.15 ± 0.77	0.971		
Psychological capital	97.59 ± 14.06	4.88 ± 0.70	0.970	0.770	0.930
Self‐efficacy	29.88 ± 4.33	4.98 ± 0.72	0.922		
Hope	29.00 ± 4.49	4.83 ± 0.75	0.916		
Resilience	24.30 ± 3.66	4.86 ± 0.73	0.918		
Optimism	14.41 ± 2.82	4.80 ± 0.94	0.948		
Professional identity	37.18 ± 7.97	3.72 ± 0.80	0.947	0.861	0.949

The results showed that the professional identity of anesthesia nurses was positively correlated with transformational leadership (*r* = 0.563, *p* < 0.01) and psychological capital (*r* = 0.571, *p* < 0.01), and the psychological capital of anesthesia nurses was positively correlated with transformational leadership (*r* = 0.596, *p* < 0.01), as detailed in Table 3.

**TABLE 3:** Correlations among variables (*n* = 336).

Variables	Transformational leadership	Psychological capital	Professional identity
Transformational leadership	1		
Psychological capital	0.596^∗∗^	1	
Professional identity	0.563^∗∗^	0.571^∗∗^	1

^∗^^∗^ *p* < 0.01.

### 4.4. The Mediating Effect of Psychological Capital

**FIGURE 2:**
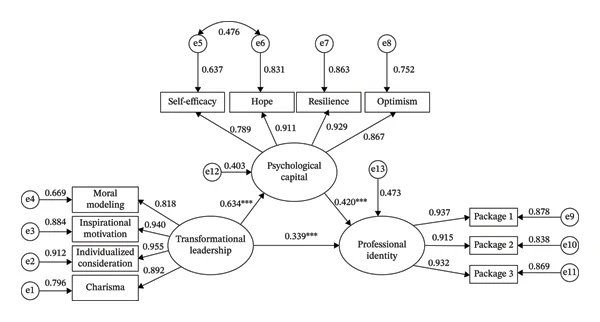
The mediating effect model of anesthesiology nurses’ psychological capital between transformational leadership and professional identity.

The structural equation model contains three latent variables: (1) Transformational leadership, whose explicit variables are the four dimensions of the transformational leadership scale; (2) Psychological capital, whose explicit variables are the four dimensions of the psychological capital scale; (3) Professional identity, whose explicit variables are the 10 items of the professional identity scale. To enhance the stability of the structural model, we applied item parceling to the professional identity dimension, following the recommendations of statistical expert Wen Zhonglin on item parceling in SEM [38]. This study employed a random parceling strategy to improve model fit. Specifically, item package 1 includes items 2, 8, and 10; item package 2 includes items 4, 5, 7, and 9; and item package 3 includes items 1, 3, and 6. The initial structural equation model was established with transformational leadership as the independent variable, professional identity as the dependent variable, and psychological capital as the mediating variable. The test results showed that the overall fit of the initial model was unsatisfactory: *χ* ^2^/df = 3.909, RMSEA = 0.093, GFI = 0.919, CFI = 0.970, NFI = 0.960, TLI = 0.960. The parameters were estimated by maximum likelihood using AMOS 28.0 software, and the model was modified (by adding a correction path between e5 and e6). The revised model fitting results showed that: *χ* ^2^/df = 2.909, RMSEA = 0.075, GFI = 0.943, CFI = 0.981, NFI = 0.971, TLI = 0.974, all meet the standards, and the model fits well. The revised structural equation model is shown in Figure 2.

Using the bootstrap method for 5000 repeated samples, with a confidence interval of 95% and excluding 0, we tested the mediating effect. The results indicated that the total effect of transformational leadership on anesthetic nurses’ professional identity was significant (*β* = 0.606, *p* < 0.001). The direct effect was 0.339, accounting for 55.94% of the total effect. The mediating effect of psychological capital was 0.267, accounting for 44.06% of the total effect, and the 95% confidence intervals for both the direct and mediating effects did not include 0. Therefore, both the direct and indirect paths reached a significant level, indicating that psychological capital plays a partial mediating role in the relationship between transformational leadership and the professional identity of anesthetic nurses. Based on this, both Hypothesis 1 (transformational leadership is significantly positively correlated with professional identity) and Hypothesis 2 (psychological capital has a mediating effect) were verified. Details are presented in Table 4.

**TABLE 4:** The mediation analysis of psychological capital between transformational leadership and professional identity.

Effect	β	SE	95% CI		*p*	Relative effect value (%)
			Lower	Upper		
Direct effect TFL ⟶ PI	0.339	0.096	0.139	0.516	0.001	55.94
Indirect effect TFL ⟶ PsyCap ⟶ PI	0.267	0.063	0.155	0.399	0.001	44.06
Total effect	0.606	0.055	0.486	0.701	< 0.001	100.00

*Note:* TFL: transformational leadership.

Abbreviations: CI, confidence intervals; PI, professional identity; PsyCap, psychological capital; SE, standard error.

## 5. Discussion

The level of professional identity among anesthesia nurses is a key factor influencing the quality of clinical work and promoting professional development. This study aimed to examine the relationships among transformational leadership, psychological capital, and professional identity among anesthesia nurses. The findings reveal that transformational leadership is significantly associated with professional identity, both directly and indirectly through psychological capital, and that all proposed hypotheses are supported.

### 5.1. The Professional Identity of Anesthesia Nurses in China

This study indicated that the professional identity scale for anesthesia nurses scored 37.18 (SD = 7.97), with an average item score of 3.72 (SD = 0.80), which was moderate and consistent with findings reported for general nurses [39]. Several factors may explain this result. First, the unique work environment and professional culture of the anesthesia nurse’s department. Anesthesia nursing work is predominantly concentrated in fast‐paced clinical environments such as operating rooms and postanesthesia care units, characterized by high technicality, task orientation, and efficiency priority. This has gradually formed a specific culture marked by relatively depersonalized interpersonal relationships, which diminishes nurses’ perception of their professional emotional labor and value, thereby affecting the construction of their professional identity [8]. Furthermore, significant overlaps exist between the work scopes of anesthesia nurses and anesthesiologists, blurring team role boundaries and hindering the articulation of anesthesia nursing’s unique value. Simultaneously, anesthesia nurses perform their duties within the scope of anesthesiologists’ work, with their responsibilities fluctuating based on factors like anesthesiologists’ professional habits and workload. This variability makes it difficult for anesthesia nurses to establish stable expectations of their professional identity [40]. Second, there is currently a phenomenon of “training‐heavy and utilization‐light” in the human resources of anesthesia nursing in China [41], where talent development and practical application are disconnected, hindering the manifestation of professional value. Furthermore, in team collaboration, the professional value of anesthesia nurses remains underappreciated. Doctors within the same department have skewed perceptions of their contributions, and nurses from other departments lack understanding of the connotation and practical boundaries of their practice [42, 43]. Consequently, it was difficult for anesthesia nurses to obtain due understanding and empathy in professional interactions. In summary, it is recommended that managers should: (1) Clarify the professional positioning of anesthesia nurses. Develop standardized job descriptions based on the actual work of anesthesia nursing and refine the responsibility lists for nurses and doctors. Delineate task allocation and collaboration processes to institutionally resolve role ambiguity and fluctuating responsibilities. (2) Optimize anesthesia nurses’ training and utilization methods. Closely align talent development with job competency requirements. Meanwhile, goal‐oriented performance evaluations and policy incentives should be adopted to fully activate nurses’ initiative, as well as to ignite their professional passion and sense of existence. (3) Build a diversified communication platform. Organize cross‐department communication to enhance peer understanding. Optimize the collaboration model between medical staff, establish an internal peer review system with departments, and guide anesthesiologists to recognize the importance of anesthesia nursing in teamwork.

### 5.2. Relationship Among Transformational Leadership, Psychological Capital and Professional Identity

In this study, the mean score on the transformational leadership scale was 107.63 (SD = 18.96), indicating an upper‐middle level and aligning with findings reported for general nurses [44]. Among the subscales, moral modeling received the highest score. This finding aligns with Chinese traditional culture, which places great importance on moral ethics. By upholding strong professional ethics, leading by example, and setting a positive standard, nursing managers can provide anesthesia nurses with guidance on values and behavioral models. The study also identified a significant positive correlation between transformational leadership and the professional identity of anesthesia nurses, thereby supporting H1 and corroborating prior research among teachers [45], further verifying the promoting effect of transformational leadership on professional identity. Transformational leaders promote the integration of personal values with the organizational vision by focusing on nurses’ individual needs and providing ample resources for professional development [46], which is particularly crucial for Chinese anesthesia nurses during the critical stage of professional identity formation. Meanwhile, managers who are adept at utilizing transformational leadership styles are themselves exemplary resources that can be emulated [47]. These exemplary resources serve as a source of energy that motivates anesthesia nurses to engage in positive professional behaviors, such as proactively improving their professional skills and setting new goals. Anesthesia nurses view the moral modeling, inspirational motivation, and individualized consideration of transformational leaders as valuable initial resources. They actively preserve these through recognition, emulation, and follow‐through, transforming them into core capital for their professional development. These practices help anesthesia nurses clarify their professional roles, appreciate the value of their work, and strengthen their professional identity in practice.

This study revealed a significant positive correlation between psychological capital and professional identity, consistent with previous research findings among nursing students [48]. For anesthesia nurses, occupational characteristics such as high work intensity, substantial responsibility, and complex interpersonal relationships are prone to resource depletion [49]. According to COR theory, individuals have an intrinsic motivation to protect their existing resources and avoid resource depletion. Psychological capital, as a core internal resource of individuals, helps individuals achieve this goal and further promotes positive career outcomes. When facing work‐related difficulties and pressure, anesthesia nurses with higher levels of psychological capital tend to maintain an optimistic and positive attitude, actively adopting coping strategies rather than passively retreating (the latter often resulting in resource loss). Through this process, they actively maintain existing resources and reduce resource consumption by actively improving their professional abilities and enhancing work engagement. At the same time, their professional identity is constantly strengthened in this process of resource maintenance and self‐improvement [50, 51]. Resilience, an important component of psychological capital, refers to an individual’s ability to gather, select, and utilize resources to effectively respond to psychological stress [52]. Nurses with high resilience are not immune to stress; rather, they possess a stronger ability to identify and leverage available resources to overcome adversity or restore equilibrium following setbacks [53], thereby mitigating the risk of resource loss. This process helps nurses reduce negative emotional reactions through their inner strength, thereby lowering the likelihood of a professional identity crisis [54, 55]. By successfully coping with work‐related stress, nurses can enhance their sense of professional accomplishment and self‐efficacy, ultimately consolidating and strengthening their professional identity [56]. Notably, for nurses with low psychological capital, prolonged resource depletion without adequate replenishment can precipitate emotional exhaustion, which may weaken or even cause the collapse of their professional identity.

This study demonstrated a significant positive correlation between transformational leadership and psychological capital, a finding that corroborates prior research on corporate employees [57]. According to COR theory, the acquisition of initial resources increases an individual’s ability and opportunities to acquire further resources. In this study, transformational leaders provided anesthesia nurses with clear goal orientation and emotional support in high‐pressure, high‐risk work environments through behaviors such as fostering a positive work environment, offering cognitive and social support, and clearly communicating a professional vision [58, 59]. Once nurses acquire these initial resources, they are more likely to engage in resource‐investing behaviors (such as proactive learning and seeking challenging tasks), which in turn lead to the acquisition of new resources (such as skill enhancement, peer recognition, and experiences of personal growth), ultimately accumulating into stable psychological capital. It is worth noting that whether transformational leadership can effectively promote psychological capital among anesthesia nurses also depends on the nurses’ perception of the alignment between leadership resource provision and their own developmental needs. For example, for newly hired anesthesia nurses, transformational leadership helps them quickly adapt to their roles through emotional support and clear clinical guidance, thereby enhancing their self‐efficacy and optimism and solidifying the foundation of their psychological capital. For senior nurses, their perceived transformational leadership is more reflected in reasonable authorization and career development empowerment, which effectively stimulates their sense of career hope and resilience, helps them achieve sustained growth in complex clinical anesthesia cooperation, and further enhances their psychological capital. This differentiated approach to resource provision enables the resources provided by leadership to be more effectively transformed into stable psychological capital for nurses, thereby reinforcing the positive association between transformational leadership and psychological capital.

### 5.3. Mediating Role of Psychological Capital

This study revealed that psychological capital partially mediates the relationship between transformational leadership and professional identity among anesthesia nurses. The mediating effect was 0.267, accounting for 44.06% of the total effect, which confirms H2. Based on the value‐added spiral inference from COR theory [17], anesthesia nurses regard value guidance, empowerment motivation, individualized consideration, and other aspects of transformational leadership as valuable external resources. To effectively preserve and continuously increase these resources, their professional behaviors tend to be more positive and proactive. This process elevates their self‐efficacy and work resilience, thereby strengthening psychological capital as an internal resource. High levels of psychological capital help nurses maintain strong adaptability when facing anesthesia crises or team conflicts, enabling them to derive professional experience and a sense of value from these situations, ultimately enhancing their professional identity. This pathway reveals the mediating mechanism from external leadership resources to individual psychological capital and finally to the construction of professional identity. Conversely, when lacking external support from transformational leadership, individuals face insufficient existing resources or even the risk of further resource depletion. To replenish resources or prevent further depletion, nurses must continuously mobilize their limited psychological resources. Their ability to effectively integrate and use resources to solve professional challenges is hampered by this prolonged depletion, which ultimately depletes psychological capital and undermines their professional identity [60]. Therefore, transformational leadership significantly influences the professional identity of anesthesia nurses through psychological capital.

## 6. Implications for Nursing Administration and Practice

Based on the COR theory, this study proposes suggestions for enhancing the professional identity of anesthesia nurses through both external resource supply and internal psychological resource cultivation. First, anesthesia nursing managers should optimize their leadership style by adopting positive approaches (e.g., transformational leadership) to provide supportive external resources for anesthesia nurses at the organizational level. Specifically, this includes fully empowering nurses to actively develop their professional skills; providing individualized consideration that effectively integrates individual growth with organizational vision; and emphasizing leaders’ moral example and value guidance, providing anesthesia nurses with spiritual strength and role model resources. Second, managers should closely monitor the psychological capital of anesthesia nurses. It is recommended that nursing managers systematically create an optimistic work environment. On this basis, effective approaches should be adopted to cultivate and develop the psychological capital of anesthesia nurses, such as assigning competent and challenging tasks to hone their work resilience, experiencing successful experiences, and enhancing their self‐efficacy. Furthermore, anesthesia nurses should develop strong coordination and management skills to optimize and integrate both internal and external resources, thereby supporting their individual career development.

## 7. Limitations

This study has several limitations. First, all variables were measured using self‐reported data collected at the same time point, which may pose a potential risk of CMB. Although this study employed a single‐factor CFA to test for this bias, this method is only one of many available methods and has inherent limitations. More critically, regardless of the statistical test results, the cross‐sectional self‐report design itself inevitably carries the risk of CMB. Therefore, we cannot completely rule out the possibility that the observed associations between variables may be partially exaggerated or biased due to common method variance. Second, as a cross‐sectional survey, it is not possible to dynamically assess changes in the professional identity of anesthesia nurses at different stages of career development, nor can it establish causal relationships among the variables, only revealing relevant associations. Regarding the above two points, future research could adopt multisource data collection methods (such as supervisors’ or colleagues’ evaluations) or a longitudinal repeated measurement design to draw more reliable and causal conclusions. Finally, convenience sampling was used in this study. Although the sample spanned nine provinces and municipalities′ tertiary hospitals, it was mainly concentrated in the developed areas of the eastern and central regions, with insufficient coverage in the underdeveloped western regions, limiting the generalizability of the conclusions. It is worth noting that 88.7% of the sample were female, which is consistent with the gender composition of nursing staff in China and reduces the potential impact of gender bias. Future research is recommended to include more institutions from underdeveloped western regions and healthcare facilities at various levels to enhance the universality of the study’s conclusions.

## 8. Conclusions

This study elucidates the relationship among transformational leadership, psychological capital, and professional identity based on the COR theory. Transformational leadership was positively associated with anesthesia nurses’ professional identity directly and also had an indirect associative effect on professional identity through psychological capital. We encourage managers to actively adopt transformational leadership practices as a strategic approach to strengthen nurses′ professional identity. At the same time, managers should pay attention to the psychological capital of anesthesia nurses, cultivate and stimulate their inherent resources, and leverage their role in promoting professional identity.

## Author Contributions

Rui Gao: conceptualization, methodology, investigation, data curation, software, formal analysis, and drafting the manuscript. Peishuang Wang: investigation, methodology, and data curation. Fang Zhou and Jiao Wu: review and editing. Nana Liu and Huihui Hu: validation, project administration, funding acquisition, and review and editing.

## Funding

This study was funded by Postgraduate Research & Practical Innovation Program of Jiangsu Province (SJCX24_1558) and Research Innovation Plan of the School of Nursing, Xuzhou Medical University (HLYC202506).

## Disclosure

All authors reviewed and approved the final version of the manuscript.

## Conflicts of Interest

The authors declare no conflicts of interest.

## Data Availability

The data that support the findings of this study are available on request from the corresponding author. The data are not publicly available due to privacy or ethical restrictions.
